# mTORC1 Is Essential for Early Steps during Schwann Cell Differentiation of Amniotic Fluid Stem Cells and Regulates Lipogenic Gene Expression

**DOI:** 10.1371/journal.pone.0107004

**Published:** 2014-09-15

**Authors:** Andrea Preitschopf, Kongzhao Li, David Schörghofer, Katharina Kinslechner, Birgit Schütz, Ha Thi Thanh Pham, Margit Rosner, Gabor Jozsef Joo, Clemens Röhrl, Thomas Weichhart, Herbert Stangl, Gert Lubec, Markus Hengstschläger, Mario Mikula

**Affiliations:** 1 Institute of Medical Genetics, Medical University of Vienna, Vienna, Austria; 2 Department of Pediatrics and Adolescent Medicine, Medical University of Vienna, Vienna, Austria; 3 1st Department of Obstetrics and Gynaecology, Semmelweis University Medical School, Budapest, Hungary; 4 Institute of Medical Chemistry, Medical University of Vienna, Vienna, Austria; INSERM, France

## Abstract

Schwann cell development is hallmarked by the induction of a lipogenic profile. Here we used amniotic fluid stem (AFS) cells and focused on the mechanisms occurring during early steps of differentiation along the Schwann cell lineage. Therefore, we initiated Schwann cell differentiation in AFS cells and monitored as well as modulated the activity of the mechanistic target of rapamycin (mTOR) pathway, the major regulator of anabolic processes. Our results show that mTOR complex 1 (mTORC1) activity is essential for glial marker expression and expression of Sterol Regulatory Element-Binding Protein (SREBP) target genes. Moreover, SREBP target gene activation by statin treatment promoted lipogenic gene expression, induced mTORC1 activation and stimulated Schwann cell differentiation. To investigate mTORC1 downstream signaling we expressed a mutant S6K1, which subsequently induced the expression of the Schwann cell marker S100b, but did not affect lipogenic gene expression. This suggests that S6K1 dependent and independent pathways downstream of mTORC1 drive AFS cells to early Schwann cell differentiation and lipogenic gene expression. In conclusion our results propose that future strategies for peripheral nervous system regeneration will depend on ways to efficiently induce the mTORC1 pathway.

## Introduction

Specialized glial cells, known as Schwann cells, are essential for correct development as well as maintenance of the peripheral nervous system (PNS) [1]. Most importantly, Schwann cells are needed for regeneration and repair of nerve lesions, because in case of nerve damage, glial cells remyelinate regenerating axons and guide the growing axons to their targets [2], [3], [4]. However, adult Schwann cells are hardly available for cell-based regeneration approaches due to strong donor site morbidity after cell isolation and due to their slow *in vitro* proliferation characteristics.

Therefore, amniotic fluid stem (AFS) cells are candidates as a novel stem cell source for Schwann cell differentiation. Since the discovery of Oct4-positive cells within human amniotic fluid [5], several studies have reported the broadly multipotent potential of these cells [6], [7], [8], [9]. Immunoselection for c-kit has been shown to be sufficient to yield cells which have the potential to differentiate towards adipogenic, osteogenic, myogenic, endothelial, hepatic and neurogenic lineages [10]. Importantly, c-kit-selected AFS cells can be grown continuously in culture maintaining a stable karyotype and exhibiting high proliferative capacity [10], [11]. While mesenchymal stem cells from the bone marrow of rats and humans were successfully differentiated towards Schwann cells [12], [13], it is currently unknown whether also monoclonal human c-kit and Oct4-positive immuno-selected AFS cells harbor the potential to give rise to Schwann cells.

During the course of early development, Schwann cells not only express lineage restricted differentiation markers such as nerve growth factor receptor (NGFR), glial fibrillary acidic protein (GFAP) and S100b, but also up-regulate lipogenic gene expression [14], [15]. SREBP family transcription factors are the main regulators of lipogenic genes, which include the low density lipoprotein receptor (LDLR) and enzymes like HMG-CoA reductase (HMGCR) and NAD(P) dependent steroid dehydrogenase like (NSDHL) [16]. Recently, mTORC1 was suggested to be involved in SREBP activation [17] and it was shown that conditional deletion of mTOR in mice resulted in a reduced myelin production by Schwann cells and reduced nerve conduction [1]. The underlying mechanism, however, is still unclear.

In the present study we investigated whether monoclonal human AFS cells can be used to generate early Schwann cells and analyzed the role of mTORC1 during this process. We applied a novel protocol to differentiate Schwann cells from AFS cells and demonstrated that inhibition of mTORC1 efficiently blocks Schwann cell differentiation, whereas induction of lipogenic genes stimulated Schwann cell differentiation.

## Materials and Methods

### Cells, cell culture of human AFS cells

The monoclonal human amniotic fluid stem (AFS) cell line Q1 and a high Oct4 expressing single cell clone derived from the CD117/2 population was used in the study [10], [11], [18]. Cells were maintained in α-MEM (Gibco-Invitrogen, USA) supplemented with 15% Fetal Bovine Serum (HyClone, USA), 18% Chang B, 2% Chang C (Irvine Scientific, USA), 2.5 mM L-Glutamine (PAA, Austria), 50 mg/L streptomycin sulphate (PAA, Austria) and 30 mg/L penicillin (PAA, Austria). For neural crest marker expression melanoma-derived MCM1 cells were used as positive control [19]. All cells were cultivated at 37°C in 5% CO_2_.

### Differentiation of human AFS cells into a Schwann cell phenotype

To initiate human AFS cells differentiation into a Schwann cell phenotype, AFS cells were dissociated (80–90% confluence) with 0.25% trypsin/EDTA (PAA, Austria) and subsequently plated on 6 cm plastic dishes at a concentration of 10^5^/cm^2^ in media consisting of α-MEM and 1 mM β-mercaptoethanol (Sigma-Aldrich, USA). After 24 hours, media was removed, cells were washed with PBS (PAA, Austria) for 3 times, and media consisting of α-MEM, 10% Fetal Bovine Serum (PAA, Austria) and 35 ng/ml retinoic acid (Sigma-Aldrich, USA) was added. After 72 hours, cells were washed with PBS for 3 times and media was replaced with differentiation media consisting of α-MEM, 10% Fetal Bovine Serum (PAA, Austria), 20 ng/mL epidermal growth factor (EGF; Peprotech, UK), 20 ng/mL basic fibroblast growth factor (bFGF; Peprotech, UK), 5 mM forskolin (Sigma-Aldrich, USA), 5 ng/mL platelet-derived growth factor-AA (PDGF-AA; Peprotech, UK) and 200 ng/mL recombinant human heregulin-beta1 (HRG; Peprotech, UK). Media were additionally supplemented with 2.5 mM L-Glutamine, 50 mg/L streptomycin sulphate and 30 mg/L penicillin. Differentiation media was changed every 3 days. Cells were cultivated at 37°C in 5% CO_2_. The cells were incubated for another 10 days under these conditions and then harvested for further investigation.

### Animals

7 week-old C57BL/6 mice were treated daily with solvent control (2.5% DMSO in H_2_O) or the mTORC1 inhibitor everolimus (5 mg/kg body weight) for 4 weeks by oral gavage (n = 6 for the solvent control, n = 7 for everolimus treatment). Everolimus was a kind gift of Novartis. The animal study protocol was performed in accordance with national laws and guidelines and was approved by the Medical University of Vienna's Institutional Review Board (BMWF-66.009/0304-II/3b/2013). All animal sacrifice was performed under ketamine/rompun anesthesia, and all efforts were made to minimize suffering and the number of animals used.

### Transient transfection for gene overexpression

The HA-S6K1-RR construct used in this study was purchased via Addgene (Cambridge, MA, USA): HA-S6K1-F5A-E389-R3A, rapamycin-resistant/constitutively active. The differentiated AFS cells on day 15 were transfected with HA-S6K1-RR plasmid using Lipofectamine 2000 transfection reagent (Invitrogen, USA) and cells were kept in differentiation media for another 72 hours.

### Immunofluorescence staining

Cells cultured on chamber slides or 48-well plate were fixed in 4% (w/v) paraformaldehyde at room temperature for 30 min. After fixation, cells were treated with PBS containing 0.1% Triton X-100 for 5 min at room temperature and then blocked with PBS containing 1% BSA for 30 min. Subsequently cells were incubated with primary antibodies diluted in PBS containing 1% BSA overnight at 4°C. The following antibodies were used: anti-NTR-p75 (1∶200; Santa Cruz Biotechnology, USA), anti-Glial Fibrillary Acidic Protein (GFAP, 1∶200; Santa Cruz Biotechnology, USA), anti-S100b (1∶10,000; Dako, Denmark), anti-nestin (1∶500; Chemicon, Temecula, CA, USA), anti-S6 ribosomal protein phosphorylated at S240/244 (1∶1,000, Cell Signalling), anti-LDLR (1∶200; Santa Cruz Biotechnology, USA), anti-HMGCR (1∶200; a gift from Herbert Stangl). Subsequently cells were washed and incubated with the secondary antibodies Alexa Fluor 546 goat anti-mouse IgG (1∶1,000; Invitrogen, USA) or Alexa Fluor 546 goat anti-rabbit IgG (1∶1,000; Invitrogen, USA) at room temperature for 1 hour. For visualizing nuclei, cells were stained with 6 diamidino-2-phenylindole dihydrochloride (DAPI, Sigma-Aldrich, USA). The negative controls were generated by incubating with isotype specific control antibodies and omitting the first-step antibodies used in each experiment. Cells were observed using a fluorescence microscope (Zeiss, Germany). Quantification of immunofluorescence staining was performed by two independent researchers who were blinded regarding experimental details. A minimum of 250 cells per experiment were evaluated and cells with a staining intensity stronger than the isotype control stain were regarded as positive.

### Histology

Sciatic nerves were isolated from C57BL/6 mice, fixed with 4% (w/v) formaldehyde, dehydrated and embedded in paraffin. 4 µm sections were used for Luxol fast blue staining by incubating hydrated sections in 0.1% Luxol fast blue, 95% ethyl alcohol and 0.5% acetic acid at 56°C for 16 hours. The program ImageJ was used to manually quantify 5 representative Luxol fast blue stained cross sections per animal [20]. For myelin thickness the distance between the inner and outer myelin diameter were measured and for axonal packing the distance between the myelin wraps were traced. A minimum of 100 myelinated axons were quantified per animal. Immunohistochemistry was performed using modified citrate buffer, pH 6.1 (Dako), for 20 min at 120°C for antigen unmasking and 1% H_2_O_2_ for 15 min at room temperature to quench endogenous peroxidases. Then the slides were blocked in PBS containing 1% BSA for 20 min at room temperature and slides were incubated with antibodies for S100b (1∶10,000; Dako, Denmark), nestin (1∶500; Chemicon, Temecula, CA, USA) and S6 ribosomal protein S240/244 (1∶1,000, Cell Signalling) over night at 4°C and subsequently incubated with biotinylated secondary antibodies and avidin biotin complexes conjugated with peroxidase (Jackson Lab, West Grove, PA, USA) for 45 min at room temperature. Aminoethyl carbazole (AEC, Dako) was used to visualize the staining (20 min) and the slides were counterstained with hematoxylin.

### RNA extraction and reverse transcription polymerase chain reaction (RT-PCR) analysis of gene expression

Total RNA was extracted from either the mouse sciatic nerve tissue or the AFS cells at different time points of differentiation (day 0, 5, 8, 15) and conditions with RNeasy Mini Kit (Qiagen). cDNA was synthesized from 5 µg total RNA using GoScript Reverse Transcription System (Promega) according to the manufacturer's instructions. Real-time (SYBR Green) PCR was performed using a CFX96 TouchTM Real-Time PCR Detection System (Bio-Rad) with the following cycle conditions: 95°C for 2 min, 40 cycles at 95°C for 15 s, 60°C for 1 min. 5 µl GoTaq q PCR Master Mix (Promega) and 1 µl cDNA for each reaction in final volume of 10 µl were used. Human primers were used for human AFS cells and mouse primers were used for the mouse sciatic nerve tissue in RT-PCR. The sequences of primers used were as follows: for human S100b: Forward 5′- CGA ACT GAA GGA GCT CAT CAA CAA-3′, Reverse 5′-AAC TCG TGG CAG GCA GTA GTA ACC-3′. For human nestin: Forward 5′-AGC AGG AGA AAC AGG GCC TAC AG-3′, Reverse 5′-CTG AAA GCT GAG GGA AGT CTT GGA-3′. For human SREBF1: Forward 5′-CGT CTC CTT GGT GCT TCT CTT TGT-3′, Reverse 5′-GAT GAG GTT CCA GAG GAG GCT ACA-3′. For human SREBF2: Forward 5′-ACT CTG AGC CAG GAA GCC CTC TAT-3′, Reverse 5′-CAG AAC CTG ACT CGA ATG ACA GGA-3′. For human NSDHL: Forward 5′-TCT TCC CAT TTC CAA TCA CGA ACT-3′, Reverse 5′-TCT TCC CAT TTC CAA TCA CGA ACT-3′. For human LDLR: Forward 5′-TTT CTG GTT TCG GAG CAC GTA AAT-3′, Reverse 5′-CAG AGG CAA TAA CCC CCT ACA CAG-3′. For human HMGCR: Forward 5′-TCC AGA GCA AGC ACA TTA GCA AAG-3′, Reverse 5′-GGA CAC ACA AGC TGG GAA GAA AGT-3′. For human Beta Actin: Forward 5′-CTA TCC AGG CTG TGC TAT CCC TGT-3′, Reverse 5′-CCT TAA TGT CAC GCA CGA TTT CC-3′. For mouse S100b: Forward 5′-CCG GGC GAG AGG GTG ACA AG-3′, Reverse 5′-ACT CAT GGC AGG CCG TGG TC -3′. For mouse nestin: Forward 5′-CAG CCT CCA GGA GCG CAG AG-3′, Reverse 5′-TCA GCC TCC AGC AGA GTC CTG T-3′. For mouse SREBF1: Forward 5′-CCT GGA TTT GGC CCG GGG AG-3′, Reverse 5′-CGG GCA TCC TTC CTC AGC CC -3′. For mouse SREBF2: Forward 5′-GCC CTC TGC TGG ATG ACG CA-3′, Reverse 5′-CGG GCA TCC TTC CTC AGC CC -3′.For mouse LDLR: Forward 5′-GCC GGA GTT GCA GCA GAA GAC-3′, Reverse 5′-ACA CGG CCT CCA CAG CTG AA -3′. For mouse NSDHL: Forward 5′-GTC CCC TCC GCC GTA CAG TAA C-3′, Reverse 5′-CGT TGG CAT CCA GTA CTG CTC TCT-3′. For mouse HMGCR: Forward 5′-GGC CTC TTC GTG GCC TCC C-3′, Reverse 5′-CGC TGC TCA GCA CGT CCT CT-3′. For mouse Beta Actin: Forward 5′-AGG CAC CAG GGT GTG ATG GTG-3′, Reverse 5′-GGG CCA CAC GCA GTC CAT TG -3′. Beta Actin was used for normalization. Relative gene expression was analyzed using the comparative Ct method (2^−ΔΔCt^). All measurements were done in triplicates. Student's t-test was performed to compare the fold changes.

### Protein extraction

Cells were washed with cold PBS and harvested by rapid and gentle trypsinization at room temperature. Pellets were washed twice with cold PBS and lysed in whole cell extraction buffer containing 20 mM hepes, pH 7.9, 0.4 M NaCl, 25% glycerol, 1 mM EDTA, 0.5 mM DTT, 1 mM PMSF, 0.5 mM NaF, 0.5 mM Na_3_VO_4_ supplemented with 2 mg/ml aprotinin, 2 mg/mL leupeptin, 0.3 mg/ml benzamidinchlorid,10 mg/ml trypsin inhibitor by freezing and thawing. Supernatants were collected by centrifugation at 10000 g for 20 min at 4°C and stored at −80°C. The protein concentration of the supernatant was determined by the Bradford assay.

### Western blot

Aliquots of 10 µg of protein were denatured at 95°C for 5 min and applied on a SDS–polyacrylamide gel. Proteins separated on the gel were transferred onto PVDF membranes. For immunodetection, antibodies specific for the following proteins were used: rabbit polyclonal antibody against phospho-S6 ribosomal protein S240/244 (1∶1,000, Cell Signaling, 2215, USA) and rabbit monoclonal antibody against phospho-Akt S473 (1∶1,000, Cell Signaling, 4060, USA). Antibodies were detected using anti-rabbit IgG, an HRP-linked heavy and light chain antibody from goat (1∶10,000, Bethyl, A120-101P) according to the supplier's protocol. Signals were detected using the Pierce ECL Western Blotting Substrate (Thermo Fisher Scientific, USA) and GAPDH was used as a loading control.

### Filipin fluorescence staining in cultured cells

Cells cultured on chamber slides (Lab-Tek, Denmark) were fixed in 4% (w/v) paraformaldehyde at room temperature for 30 min. After fixation, cells were treated with 1.5 mg/ml glycine diluted in PBS for 10 min at room temperature to quench the paraformaldehyde. Afterwards cells were washed 3 times with PBS and incubated with 0.05 mg/ml filipin complex in PBS working solution (Sigma-Aldrich, USA) for 2 hours at room temperature. Cells were washed 3 times with PBS and staining was observed using fluorescence microscope (Zeiss, Germany).

### Data and statistical analysis

All experiments were performed in triplicate and representative blots are shown. Data were averaged, unless otherwise specified, and are presented as mean ± SEM. Significant differences between groups were tested by Student's unpaired t-test and p<0.05 was considered as significant.

## Results

### Differentiation of human monoclonal AFS cells towards Schwann cells

**Figure 1:**
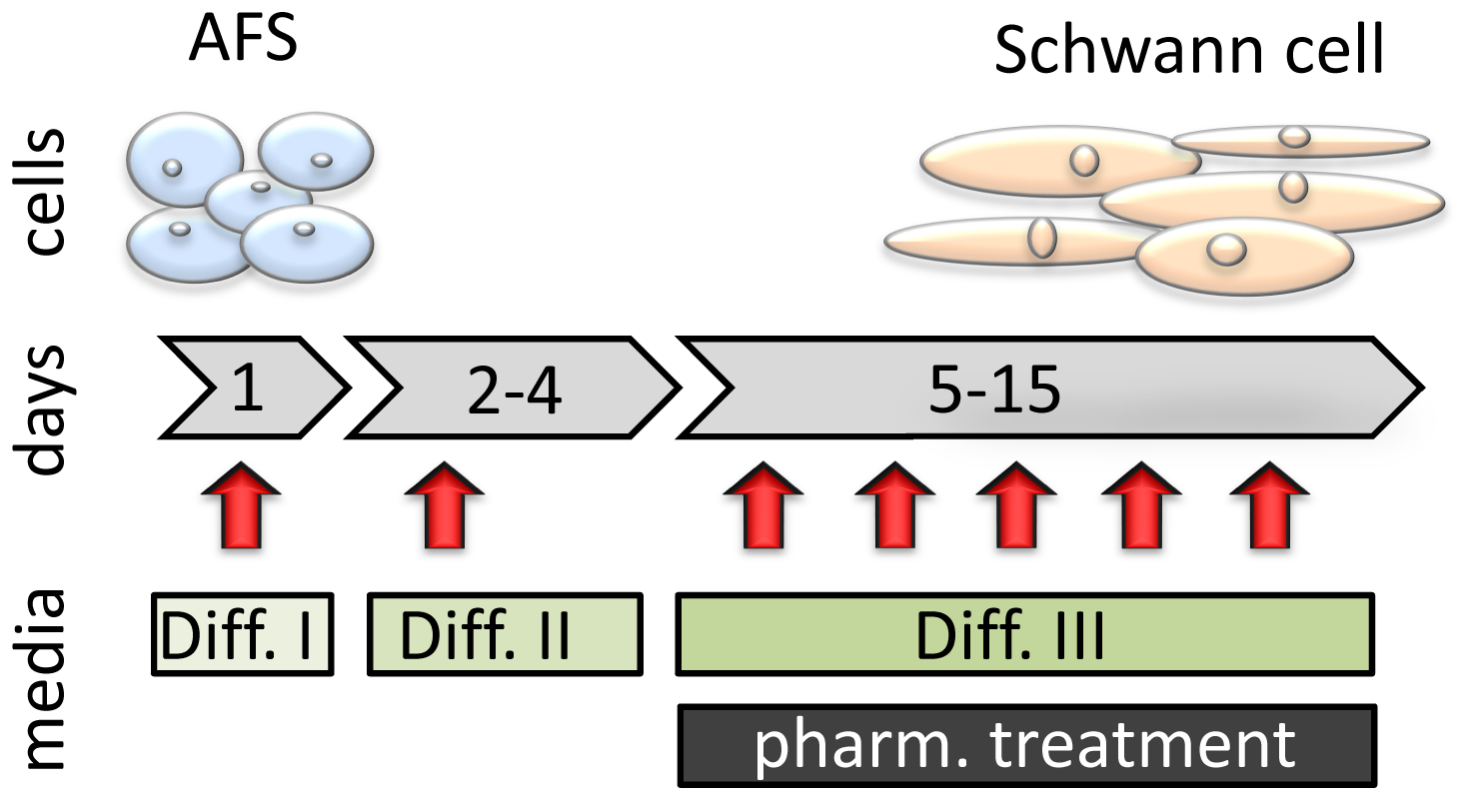
Scheme for the applied differentiation protocol. In order to initiate human AFS cell differentiation to a Schwann cell phenotype AFS cells were first treated in serum free α-MEM with 1 mM β-mercaptoethanol (Diff. I) for 24 hours. Afterwards cells were incubated in α-MEM supplemented with 10% fetal bovine serum and 35 ng/ml retinoic acid (Diff. II) for 72 hours. Subsequently, cells were cultured in α-MEM containing 10% fetal bovine serum supplemented with 20 ng/mL epidermal growth factor, 20 ng/mL basic fibroblast growth factor, 5 mM forskolin, 5 ng/mL platelet-derived growth factor-AA and 200 ng/mL recombinant human heregulin-beta1 (Diff. III) until day 15 of differentiation. Media was changed every 3 days, indicated by arrows. Pharmacologic (pharm.) treatment, consisting of rapamycin or statin, was applied together with Diff. III media.

**Figure 2:**
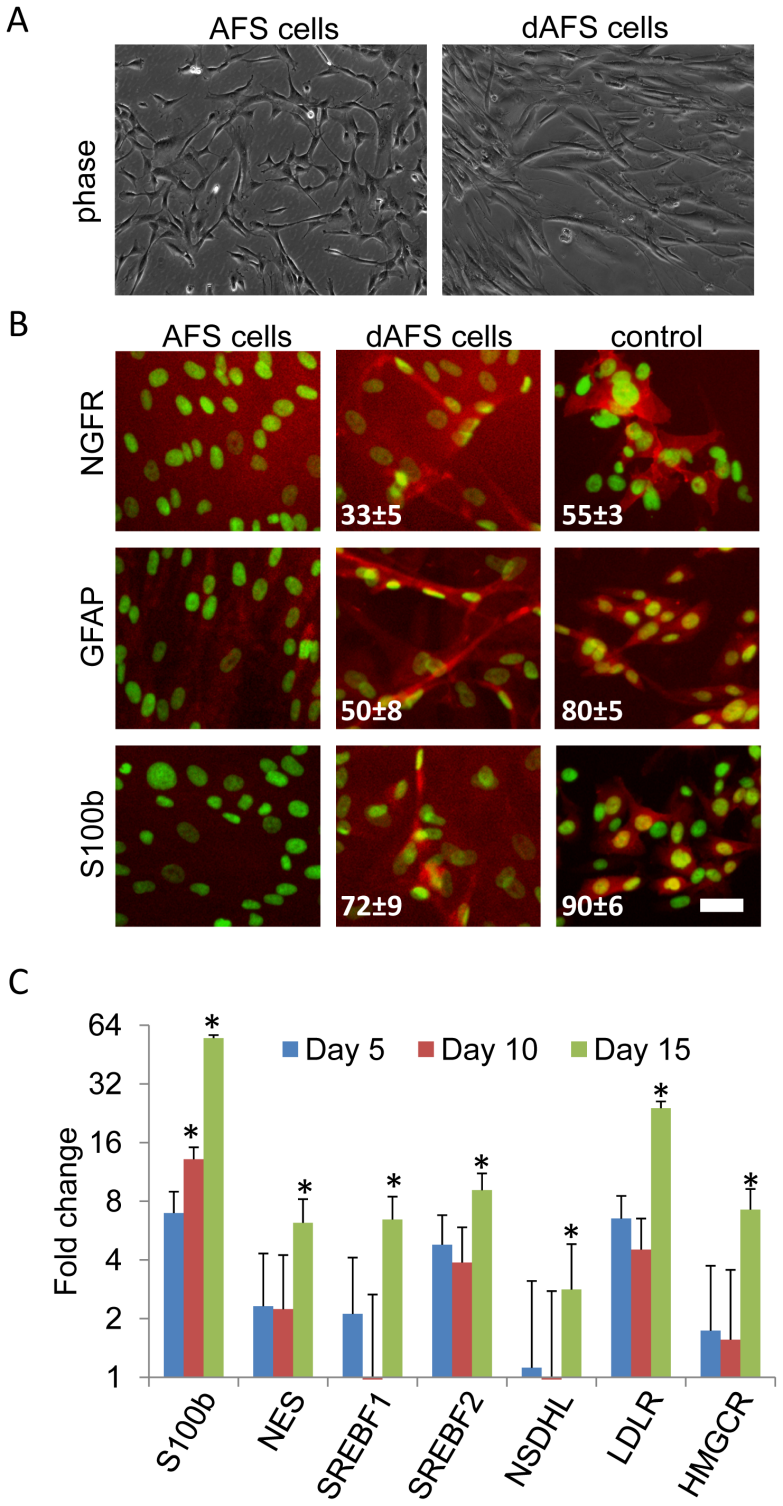
Human monoclonal amniotic fluid stem cells can be differentiated into a early Schwann cell phenotype. (A) AFS cells are small cells with omnidirectional protruding filopodia and upon differentiation to Schwann-like cells, at day 15 of treatment, cells exhibited an increase in cellular volume and an elongated cell morphology. Scale bar represents 50 µm. (B) Immunofluorescence staining of AFS cells differentiated for 15 days (dAFS) compared to undifferentiated AFS cells (AFS) and MCM1 neural crest-derived cells (control), for the Schwann cell markers NGFR, GFAP and S100b (labeled in red, nuclei labeled in green). Purity of cells is indicated as percent positive cells versus total amount of cells ± S.D. Scale bar represents 10 µm. (C) Quantitative RT-PCR of cDNA derived from AFS cells and from AFS cells subjected to Schwann cell differentiation after different time points was performed. Results are shown as fold change expression of respective genes compared to undifferentiated AFS cells. The results are expressed as means ± SEM of three independent experiments. P<0.05 for * vs undifferentiated AFS cells.

In this study, we used the previously described Q1 and CD117/2 AFS cell lines which were isolated via magnetic bead isolation selecting for c-kit positive cells [21], [22]. The Q1 cell line has been established as a monoclonal line, whereas the CD117/2 is a pool of cells. Therefore, we established single cell clones and selected the monoclonal line CD117/2-I for further studies since it showed the strongest Oct-4 expression (Fig. S1). Both AFS cell lines were used in this study to induce a pre-myelination Schwann cell phenotype via a novel three step differentiation protocol (Fig. 1). AFS cells initially displayed a uniform phenotype with a low cytoplasm to nucleus ratio and omnidirectional protruding filopodia. During 15 days of differentiation, AFS cells increased their cellular volume and displayed an elongated phenotype (Fig. 2A). To examine differentiation, we monitored expression of the established Schwann cell markers NGFR, GFAP and S100b by immunofluorescent staining and by quantitative RT-PCR (Fig. 2B and Fig. 2C).

During Schwann cell development also genes for lipid synthesis are up-regulated. This is regarded as a key element in the differentiation process, because subsequent formation of myelin is depending on the availability of lipids. SREBF1 (encoded by the isoforms Srebp1a and Srebp1c) and SREBF2 (encoded by Srebp2) are transcription factors that play a major role in cholesterol synthesis and regulate the expression of LDLR, HMGCR, and NSDHL. While HMGCR is responsible for the internal cholesterol biosynthesis, LDLR conveys cholesterol from outside into the cell [23]. NSDHL is an enzyme dependent on nicotinamide adenine dinucleotide, which functions as a sterol dehydrogenase in cholesterol synthesis [16]. Quantitative RT-PCR analysis revealed significant up regulation of these genes by day 15 of differentiation, indicating uptake as well as synthesis of cholesterol at this time point (Fig. 2C). These results suggest that monoclonal AFS cells can be differentiated to early Schwann cells by our protocol.

### Rapamycin-sensitive mTOR is critical for Schwann cell differentiation from AFS cells

**Figure 3:**
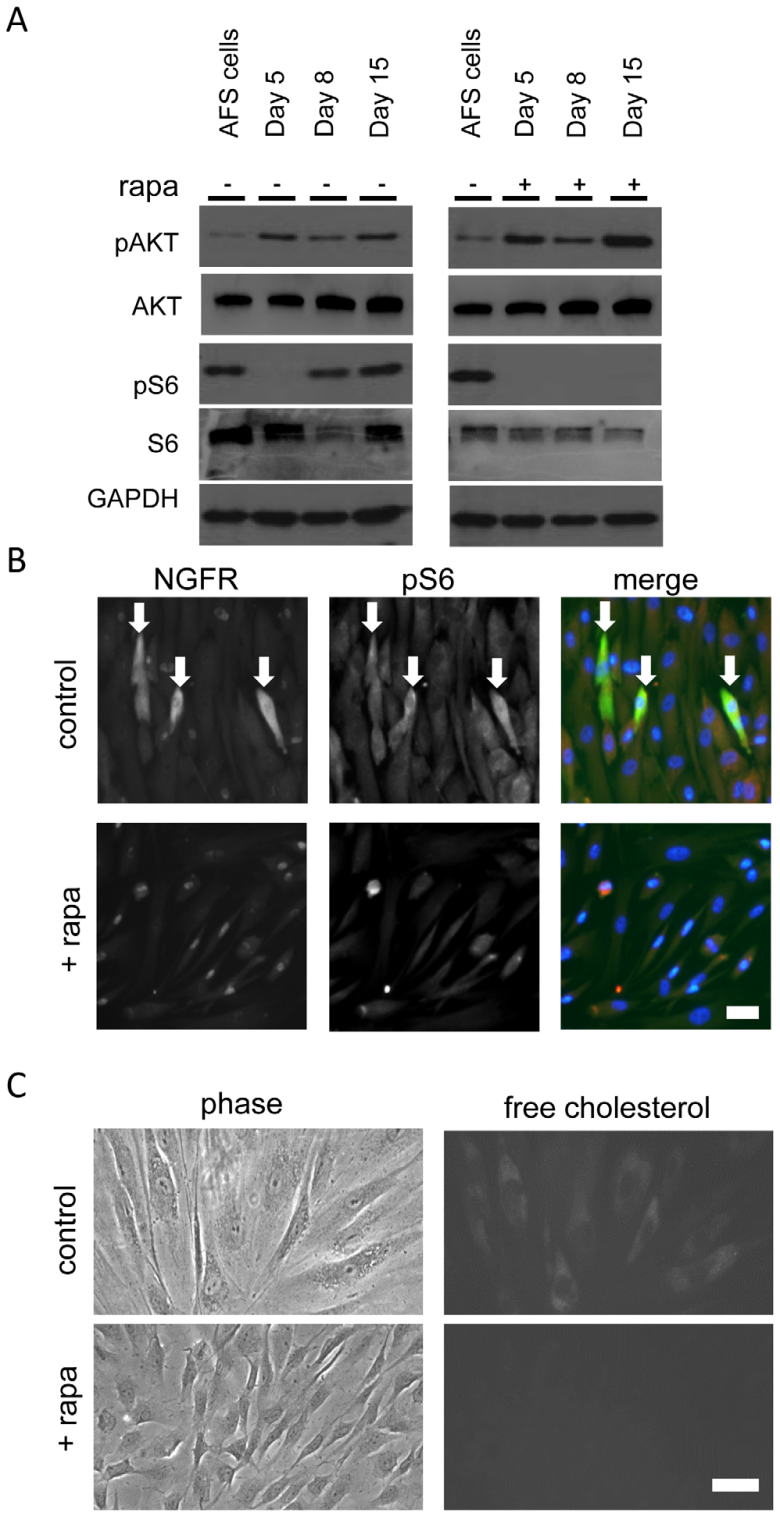
mTOR signaling is active in differentiated AFS cells and important for the differentiation process. (A) AKT phosphorylation at Ser 473 and ribosomal protein S6 phosphorylation at Ser 240/244 were quantified at the indicated time points during differentiation with and without rapamycin treatment. (B) NGFR, a marker for early differentiated AFS cells (labeled in green), was co-stained with phosphorylated S6 at Ser 240/244 protein (labeled in red) with and without rapamycin treatment (nuclei labeled in blue). Scale bar represents 10 µm. (C) Accumulation of free cholesterol was monitored by filipin III staining. Scale bar represents 10 µm.

To investigate whether mTOR signaling is involved in the regulation of human AFS cell differentiation to Schwann cells, we studied mTOR effector activation during the differentiation process. Initially AFS cells grown in Chang Medium display strong S6 phosphorylation. When differentiated, S6 phosphorylation is low on day 5 and on day 8, coinciding with a drop of total S6 protein on day 8, which was followed by a reactivation of mTOR activity until day 15 (Fig. 3A). Rapamycin, a selective mTORC1 inhibitor, was used from day 5 onwards and a concentration of 25 nM was sufficient to induce a complete block of S6 phosphorylation (Fig. 3A). In contrast, AKT phosphorylation was up-regulated compared to the undifferentiated AFS cells at all time points and rapamycin further enhanced its phosphorylation (Fig. 3A).

To analyze the functional role of mTORC1 during Schwann cell differentiation, we simultaneously examined NGFR expression and S6 phosphorylation. We detected co-expression of this Schwann cell marker in 42%±12 of all pS6 positive cells (Fig. 3B). Marker expression as well as S6 phosphorylation could be blocked completely by rapamycin treatment (Fig. 3B). Furthermore, rapamycin treatment from day 5 until day 15 of differentiation resulted in a marked decrease of the cell size as well as a decrease in the availability of free cholesterol (Fig. 3C). Importantly, rapamycin did not reduce cellular viability during long term treatment (Fig. S2). These results suggested that mTORC1 is essential for early Schwann cell differentiation.

### Schwann cell-expressed genes are down-regulated after rapamycin treatment in human AFS cells *in vitro* and in sciatic nerves from juvenile mice *in vivo*

**Figure 4:**
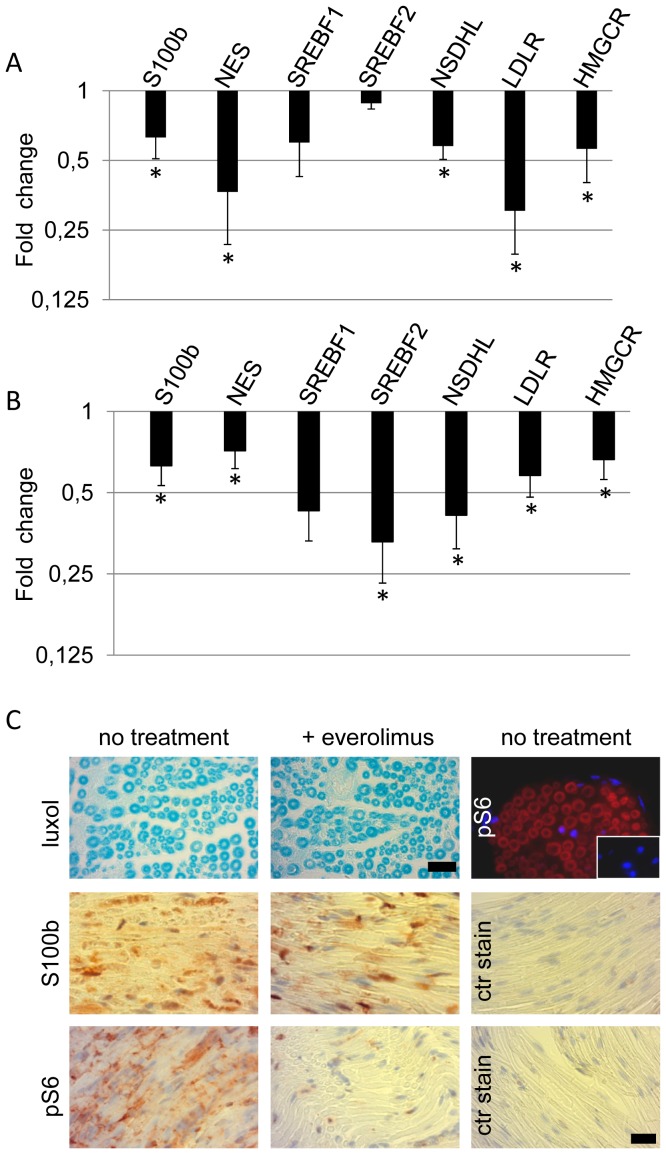
Rapamycin treatment down-regulates Schwann cell marker expression in differentiated human AFS cells and in sciatic nerves from juvenile mice. (A) Quantitative RT-PCR of cDNA derived from AFS cells differentiated towards Schwann cells for 15 days with and without rapamycin was performed to assess Schwann cell marker expression. Results are shown as fold change of respective gene expression from rapamycin-treated cells compared to control treated cells. (B) Sciatic nerves were isolated from everolimus- or control-treated mice and cDNA generated thereof was assessed for Schwann cell marker expression. Results are shown as fold change of respective gene expression from everolimus-treated mice compared to control-treated mice. The results are expressed as means ± SEM of three independent experiments. P<0.05 for * vs control treated cells or animals. (C) Sciatic nerves from untreated or treated mice were subjected to Luxol fast blue staining and immunohistochemical staining for S100b and S6 phosphorylation was performed (stained in red, nuclei in blue). Panel in upper right shows control treated sciatic nerve tissue stained for active S6 protein (red) and nuclei (blue), insert shows control antibody staining. Scale bar represents 20 µm.

To further investigate the role of mTORC1 on the regulation of human AFS cells to Schwann cell differentiation, quantitative RT-PCR analysis was performed. Continuous rapamycin treatment from day 5 onwards resulted in the down regulation of S100b and nestin at day 15 of differentiation. The transcription factors SREBF1 and SREBF2 were not significantly down-regulated during treatment, but their targets NSDHL, LDLR and HMGCR were strongly reduced (Fig. 4A).

To analyze the role of mTORC1 in Schwann cells *in vivo*, we treated 7 weeks-old mice with the mTORC1 inhibitor everolimus for 4 weeks. Schwann cells are fully myelinated by 7 weeks of age, but motor nerve conduction velocity of mice is still gradually increasing until week 10 [24]. Sciatic nerves from 7 everolimus-treated and 6 age-matched control mice were isolated and quantitative RT-PCR analysis showed a decrease of S100b and nestin (Fig. 4B). The levels of LDLR, HMGCR and NSDHL were also significantly decreased (Fig. 4B). Measuring myelin thickness and axonal packing with ImageJ on Luxol fast blue stained sections revealed no significant changes (data not shown), even though active S6 was localized within myelin containing areas and everolimus treatment efficiently blocked S6 protein phosphorylation (Fig. 4C). Additionally, expression of S100b, which resides mainly in the nucleus, was slightly reduced as visualized by immunohistochemistry (Fig. 4C).

### Lipogenic gene expression promotes human AFS to Schwann cell differentiation

**Figure 5:**
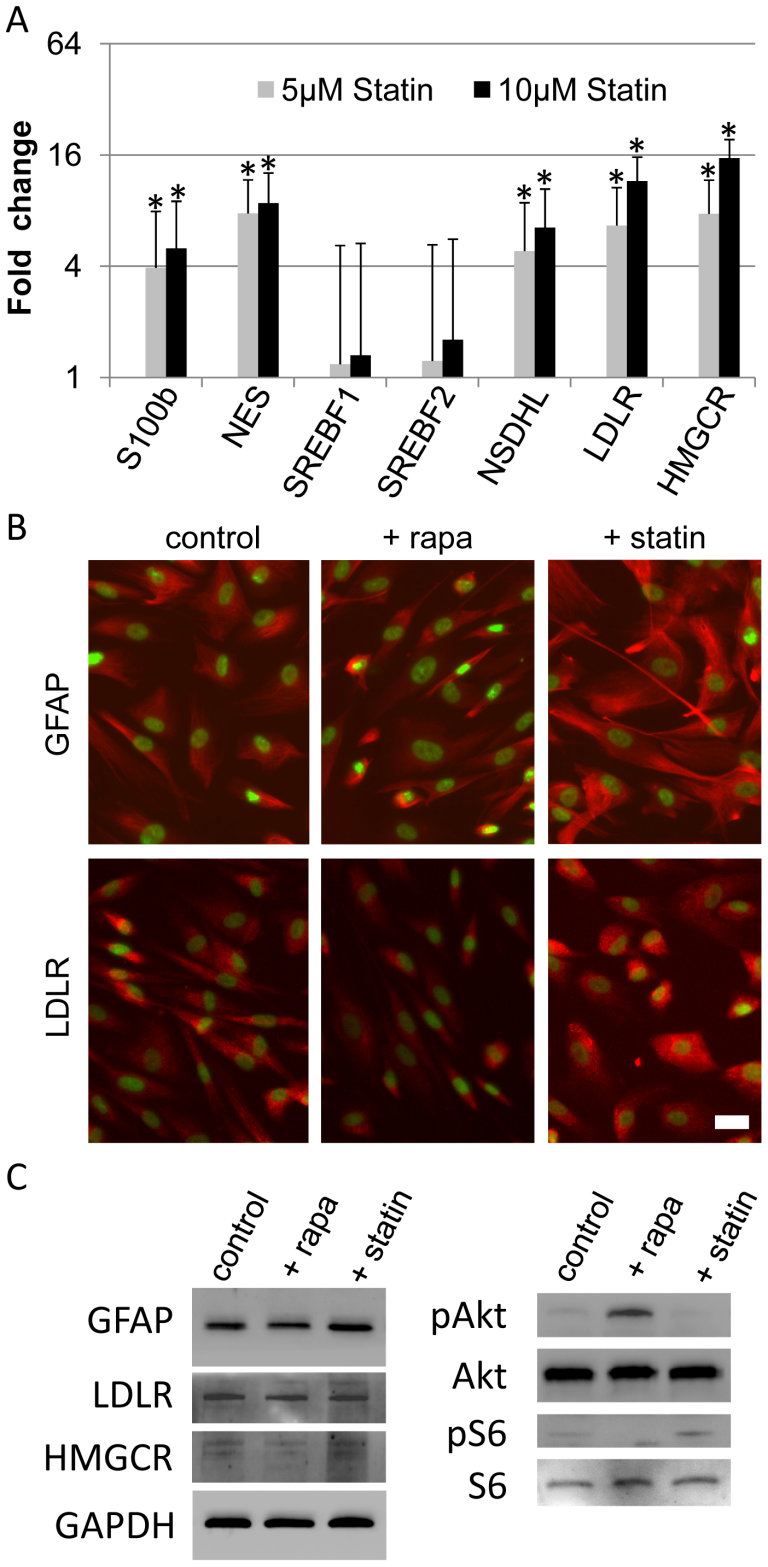
Rapamycin decreases Schwann cell markers, whereas statin induces Schwann cell markers. (A) During the last 72 hrs of differentiation, AFS cells were treated with 5 µM and 10 µM statin. After 15 days cDNA was generated and used for quantitative PCR of respective genes. The results are expressed as means ± SEM of three independent experiments. P<0,05 for * vs control treated cells. (B) AFS cells were differentiated for 15 days and since day 5 continuously treated either with 25 nM rapamycin or 1 µM of statin. Fixed cells were stained with indicated antibodies (labeled in red, nuclei in green). Scale bar represents 10 µm. (C) Western blotting of cells differentiated for 15 days and since day 5 continuously treated either with 25 nM rapamycin or 1 µM of statin. GFAP was detected at about 50 kDa, LDLR at 160 kDa and HMGCR as a double band at 90 kDa.

Next, we tested whether increasing the expression of lipogenic genes can directly influence early Schwann cell differentiation. We employed lovastatin, a competitive HMGCR inhibitor, which initially blocks cholesterol synthesis and reduces cellular cholesterol. As a consequence it promotes the activation of SREBPs, increases the expression of lipogenic genes including the LDLR and promotes LDL and cholesterol uptake in lipid-rich media [25]. In all our experiments statin treatment resulted, as expected, in the enhanced expression of LDLR, HMGCR and NSDHL mRNA (Fig. 5A) and protein (Fig. 5B, 5C and Fig. S3). Surprisingly, statin treatment until day 15 of differentiation resulted in a strong up regulation of S100b and nestin mRNA compared to control treated cells (Fig. 5A). Immunofluorescence analysis showed characteristic localization of GFAP at intermediate filament bundles and LDLR as dot like structures at the membrane and inside of cells, in control and statin treated groups (Fig. 5B). Western blotting confirmed reduced expression of the Schwann cell marker GFAP and the SREBP target LDLR upon rapamycin treatment (Fig. 5C), whereas statin treatment increased GFAP and LDLR together with an elevated phosphorylation of S6 (Fig 5C). These results suggest that the induction of lipogenic genes can enhance early Schwann cell differentiation from AFS cells.

### S6K1 promotes S100b expression, but not lipogenic genes in differentiating human AFS cells

**Figure 6:**
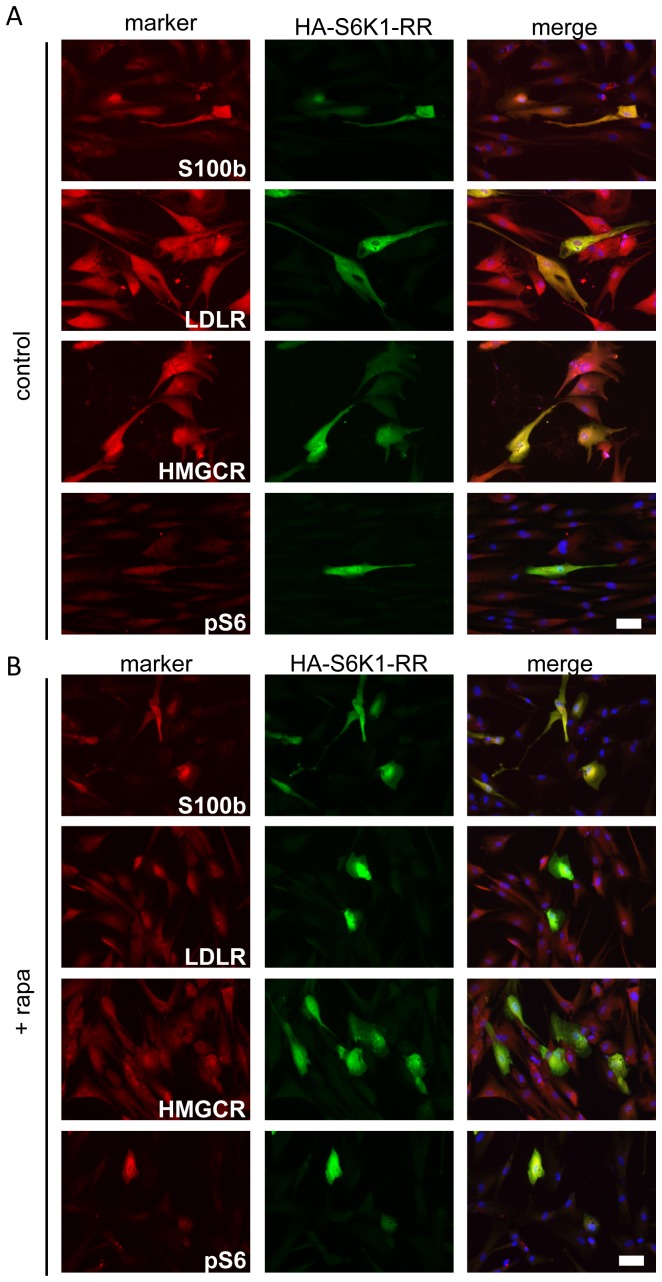
Rapamycin resistant S6K1 induces S100b, but not LDLR or HMGCR expression. (A) AFS cells were differentiated without or (B) in the presence of rapamycin and at day 15 cells were transfected with an HA-fused S6K1 rapamycin-resistant mutant (HA-S6K1-RR). After 72 hours in differentiation media containing rapamycin, cells were fixed and stained with anti-HA antibody (shown in green) combined with antibodies detecting S100b, LDLR, HMGCR and phosphorylated S6 (shown in red). Scale bar represents 25 µm.

**Figure 7:**
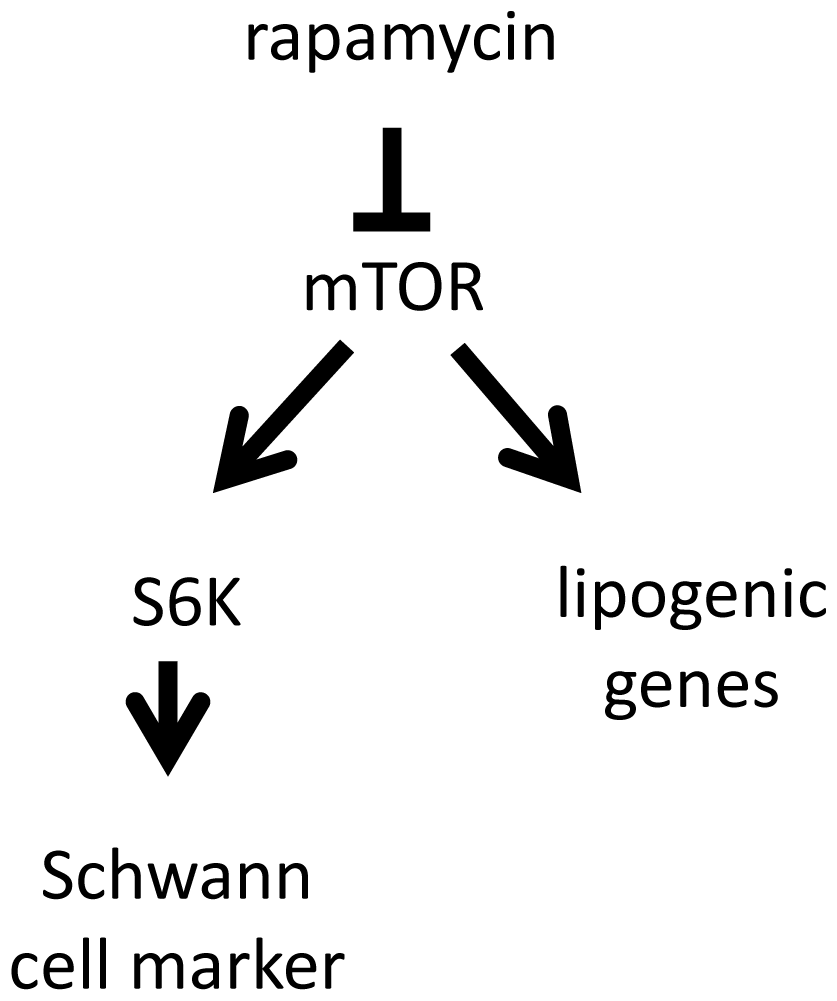
Model of mTORC1 involvement in Schwann cell differentiation. Rapamycin blocks mTORC1 and results in the down regulation of Schwann cell markers (e.g.: S100b) and in the down regulation of lipogenic genes (e.g.: LDLR, HMGCR). Our data indicates that S6K1 regulates the expression of S100b, but not of LDLR and HMGCR.

To explore pathways downstream of mTORC1 a constitutively active HA-tagged S6K1 mutant was used, which maintains its activity in the presence of rapamycin [26]. The S6K1 mutant was transfected into AFS cells differentiated for 15 days and cells were maintained in differentiation media either with or without rapamycin for 72 hours. As expected, transfected cells showed S6 phosphorylation, in contrast to un-transfected neighboring cells, indicating correct function of the expression construct (Fig. 6A, 6B lower panel). HA-positive cells re-established strong expression of S100b, the most consistently expressed Schwann cell marker available, even in the presence of rapamycin (Fig. 6A, 6B). In contrast, the lipogenic markers LDLR and HMGCR were not rescued by the S6K1 mutant in the presence of rapamycin (Fig. 6B,). We also overexpressed wild type S6K1 and detected a consistent increased expression of GFAP and NGFR, but not of nestin (Fig. S4). A TOS motive mutated S6K1 (HA-S6K1-F5A), which strongly inhibits S6 activation [27], was not able to increase S100b expression (Fig. S5). This indicates that during AFS differentiation Schwann cell-specific S100b, GFAP and NGFR are positively regulated by mTORC1 through S6K1, whereas lipogenic gene expression is dependent on mTORC1, but independent from S6K1 as summarized in Figure 7.

## Discussion

Here we analyzed early steps during monoclonal AFS cell differentiation towards Schwann cells and whether this differentiation depends on mTORC1. Cells derived from the amniotic fluid originate from the developing fetus and are therefore a mixture of different cell types. To our knowledge, here we show for the first time that c-kit selected monoclonal AFS cells can be induced by a three step protocol to express classic Schwann cell markers like NGFR, GFAP, nestin and S100b.

We cultured the cells for 15 days, which is comparable to the time period needed for human bone marrow derived mesenchymal stem cells to express Schwann cell markers [28]. We could show that this time period is sufficient to monitor SREBP target gene activation. The up regulation of lipogenic genes like NSDHL, LDLR and HMGCR recapitulates the developmental process monitored during *in vivo* Schwann cell maturation and has been shown in rats and mice [14], [29]. During post-natal development glial cells of the peripheral nervous system start to ensheath axons and hence, need to synthesize large amounts of myelin [30]. In protein lysates from sciatic nerves of new born mice strong S6 activation was shown, correlating with the time point of strongest myelin synthesis [31], [32]. 71% of the myelin membrane is composed of lipids and one of the most abundant form of lipids in the membrane is cholesterol [33]. Sterol regulatory element-binding protein, a protein necessary for SREBP processing, has been shown to be required for the myelination process, since its loss resulted in hypomyelination and abnormal gait [15]. Therefore, the induction of lipogenic genes can be considered a hallmark of functional Schwann cell development. We showed that during differentiation of AFS cells ribosomal protein S6 was phosphorylated and that this activation correlated with expression of NGFR, a prototype early Schwann cell marker. On the contrary, inhibition of S6 phosphorylation by rapamycin led to a decrease in Schwann cell marker expression, a reduction in free cholesterol accumulation and a down regulation of SREBP target genes. Rapamycin treatment of mice resulted in a decrease of Schwann cell differentiation and lipogenic marker expression on the RNA level in sciatic nerves *in vivo*. We could not detect changes in myelin composition of everolimus treated versus untreated sciatic nerves probably because myelination is already completed 7 weeks after birth. It was shown that myelin as well as overall protein translation is down regulated during maturation of peripheral nerves and that expression of the Mek1DD allel, which induces MAPK activation and also mTOR activation, can override the termination of myelin growth [32]. In this model myelinisation proceeds until P90 and treatment with rapamycin from P17 to P30 strongly reduced myelin growth and axonal packing, when compared to vehicle treated controls.

Since rapamycin treatment resulted in a suppression of SREBP target genes, which regulate both synthesis and uptake of cholesterol, we next blocked only cholesterol synthesis. Lovastatin was used to inhibit HMG-CoA reductase and, as expected, lipogenic marker genes were up regulated in response to the treatment, but surprisingly, also Schwann cell markers were enhanced. This suggests that lipid uptake, but not cholesterol synthesis is important for Schwann cell differentiation. Importantly, our results also suggest that for *in vitro* protocols, statins might promote differentiation of AFS cells or other stem cells into Schwann cells. This phenomenon could be due to the induction of LDLR expression and other lipid receptors in the presence of lipid-rich media. This enables differentiating cells to take up lipids essential for cellular homeostasis, which can support Schwann cell differentiation and may additionally induce cell signaling pathways like mTOR driven S6 kinase activation. So far, little is known on the role of LDLR for mTOR activation, but there is evidence that lipid receptors play a role during regeneration of peripheral nerves after injury [34]. Also in oligodendrocytes LDLR and VLDLR play an important role in the formation of the myelin sheath [35]. Studies have shown that statins, which up-regulate lipid receptors, are not toxic to rat Schwann cells *in vitro* and that they can induce myelin-like membranes in primary rat oligodendrocytes [36], [37]. Statins can even augment survival and differentiation of oligodendrocytes in an animal model of multiple sclerosis [38].

We rescued the rapamycin induced phenotype by overexpressing rapamycin resistant S6K1. This re-established S6 phosphorylation and led to increased Schwann cell differentiation exemplified by S100b expression. Still it could not re-establish lipogenic gene expression as demonstrated by lack of HMGCR and LDLR expression. This suggests that mTORC1 is important for the expression of Schwann cell markers and lipogenic genes, but the later are regulated independently of S6K1. Mice lacking mTOR in Schwann cells have been analyzed and they display postnatal growth retardation of myelinating Schwann cells, both radially and longitudinally [1]. Furthermore, Peterson et. al could show that mTOR directly regulates SREBP activity by controlling localization of lipin 1 [39]. These results support our finding of S6K1-independent regulation of lipogenic genes during early differentiation of AFS cells to Schwann cells.

Taken together, we have shown that rapamycin negatively regulates AFS cell differentiation to Schwann cells. We suggest that lipid uptake is an important process for efficient Schwann cell differentiation and that rapamycin-sensitive mTORC1 can regulate lipogenic gene expression independent of S6K1, whereas S6K1 activation is important for Schwann cell marker expression. Our findings propose that rapamycin, which is routinely used in clinical practice because of its immunosuppressive effects, has the potential to perturb Schwann cell function. Others have already noted that rapamycin is not at all a neuroregeneration promoting agent during studies in mice on peripheral nerve allografting [40]. We suggest that successful strategies for tissue regeneration therapy or regeneration after injury in the peripheral nervous system will depend on ways to efficiently induce the mTOR-S6K pathway. Our results further suggest statins as potential novel drugs to enhance early Schwann cell differentiation *in vitro*.

## Supporting Information

Figure S1 CD117/2 amniotic fluid stem cells were single cell cloned by limiting dilution. Single cell clone CD117/2-I displayed a normal propidium iodide stain, as observed in the starting population CD117/2. CD117/2-I exhibited a small and uniform cell morphology characteristic for bona fide amniotic fluid stem cells. Immunofluorescence staining with the santa cruz antibody sc-5789 revealed a strong nuclear Oct-4 stain in CD117/2-I cells similar to Ntera-2 carcinoembryonal cells used as control cells. Single cell clone CD117/2-A is shown as an unsuitable cell line, which displayed abnormal propidium iodide stain, heterogeneous and large cells in culture and no Oct-4 stain. Scale bar represents 5 µm. PI-FACs  =  propidium iodide fluorescence activated cell scanning.
(TIF)

Figure S2 Starting from day 5 of differentiation the effect of 25 nM rapamycin on cell viability was measured by using Alamar blue. Cells were seeded equally on day 5 of differentiation and continued to be treated with differentiation media III (see Fig. 1) either with or without the addition of rapamycin. Alamar blue was added and cells were incubated for an additional 4 hours. The fluorescence was measured at wavelengths excitation 540 nm and emission 590 nm. The average out of 4 measurments is shown +/− S.D.
(TIF)

Figure S3 AFS cells were differentiated for 15 days and continuously treated either with 25 nM rapamycin or 1 µM of statin. Fixed cells were stained with indicated antibodies (labeled in red, nuclei in green). Scale bar represents 10 µm.
(TIF)

Figure S4 AFS cells were differentiated as described in material and methods and at day 15 cells were transfected with an HA-fused wild type S6K1 (HA-S6K1) purchased from Addgene. After 72 hours in differentiation media cells were fixed and stained with anti-HA antibody (shown in green) combined with antibodies detecting Nestin, GFAP, NGFR and phosphorylated S6 (shown in red). Rapa  =  Rapamycin treatment for 72 hours. AB ctr  =  antibody control stain. Scale bar represents 25 µm.
(TIF)

Figure S5 AFS cells were differentiated as described in material and methods and at day 15 cells were transfected with an HA-fused TOS motive mutated S6K1 (HA-S6K1-F5A), purchased from Addgene. After 72 hours in differentiation media cells were fixed and stained with anti-HA antibody (shown in green) combined with antibodies detecting S100b, LDLR, HMGCR and phosphorylated S6 (shown in red). Rapa  =  Rapamycin treatment for 72 hours. AB ctr  =  antibody control stain. Scale bar represents 25 µm.
(TIF)

## References

[1] Sherman DL, Krols M, Wu LM, Grove M, Nave KA, et al (2012) Arrest of myelination and reduced axon growth when Schwann cells lack mTOR. J Neurosci 32: 1817–1825. 22302821 10.1523/JNEUROSCI.4814-11.2012 PMC4298696

[2] Martin I, Nguyen TD, Krell V, Greiner JF, Muller J, et al (2012) Generation of Schwann cell-derived multipotent neurospheres isolated from intact sciatic nerve. Stem Cell Rev 8: 1178–1187. 22664741 10.1007/s12015-012-9387-2

[3] Liang C, Tao Y, Shen C, Tan Z, Xiong WC, et al (2012) Erbin is required for myelination in regenerated axons after injury. J Neurosci 32: 15169–15180. 23100438 10.1523/JNEUROSCI.2466-12.2012 PMC3617562

[4] Ladak A, Olson J, Tredget EE, Gordon T (2011) Differentiation of mesenchymal stem cells to support peripheral nerve regeneration in a rat model. Exp Neurol 228: 242–252. 21281630 10.1016/j.expneurol.2011.01.013

[5] Prusa AR, Marton E, Rosner M, Bernaschek G, Hengstschlager M (2003) Oct-4-expressing cells in human amniotic fluid: a new source for stem cell research? Hum Reprod 18: 1489–1493. 12832377 10.1093/humrep/deg279

[6] In 't Anker PS, Scherjon SA, Kleijburg-van der Keur C, Noort WA, Claas FH, et al (2003) Amniotic fluid as a novel source of mesenchymal stem cells for therapeutic transplantation. Blood 102: 1548–1549. 12900350 10.1182/blood-2003-04-1291

[7] Prusa AR, Marton E, Rosner M, Bettelheim D, Lubec G, et al (2004) Neurogenic cells in human amniotic fluid. Am J Obstet Gynecol 191: 309–314. 15295384 10.1016/j.ajog.2003.12.014

[8] Tsai MS, Lee JL, Chang YJ, Hwang SM (2004) Isolation of human multipotent mesenchymal stem cells from second-trimester amniotic fluid using a novel two-stage culture protocol. Hum Reprod 19: 1450–1456. 15105397 10.1093/humrep/deh279

[9] Kim J, Lee Y, Kim H, Hwang KJ, Kwon HC, et al (2007) Human amniotic fluid-derived stem cells have characteristics of multipotent stem cells. Cell Prolif 40: 75–90. 17227297 10.1111/j.1365-2184.2007.00414.x PMC6496664

[10] De Coppi P, Bartsch G Jr, Siddiqui MM, Xu T, Santos CC, et al (2007) Isolation of amniotic stem cell lines with potential for therapy. Nat Biotechnol 25: 100–106. 17206138 10.1038/nbt1274

[11] Valli A, Rosner M, Fuchs C, Siegel N, Bishop CE, et al (2010) Embryoid body formation of human amniotic fluid stem cells depends on mTOR. Oncogene 29: 966–977. 19935716 10.1038/onc.2009.405 PMC8330845

[12] Brohlin M, Mahay D, Novikov LN, Terenghi G, Wiberg M, et al (2009) Characterisation of human mesenchymal stem cells following differentiation into Schwann cell-like cells. Neurosci Res 64: 41–49. 19428682 10.1016/j.neures.2009.01.010

[13] Tian X, Wang S, Zhang Z, Lv D (2012) Rat bone marrow-derived Schwann-like cells differentiated by the optimal inducers combination on microfluidic chip and their functional performance. PLoS One 7: e42804. 22880114 10.1371/journal.pone.0042804 PMC3411850

[14] Leblanc SE, Srinivasan R, Ferri C, Mager GM, Gillian-Daniel AL, et al (2005) Regulation of cholesterol/lipid biosynthetic genes by Egr2/Krox20 during peripheral nerve myelination. J Neurochem 93: 737–748. 15836632 10.1111/j.1471-4159.2005.03056.x

[15] Verheijen MH, Camargo N, Verdier V, Nadra K, de Preux Charles AS, et al (2009) SCAP is required for timely and proper myelin membrane synthesis. Proc Natl Acad Sci U S A 106: 21383–21388. 19948958 10.1073/pnas.0905633106 PMC2795508

[16] Sharpe LJ, Brown AJ (2013) Controlling cholesterol synthesis beyond 3-hydroxy-3-methylglutaryl-CoA reductase (HMGCR). J Biol Chem 288: 18707–18715. 23696639 10.1074/jbc.R113.479808 PMC3696645

[17] Bakan I, Laplante M (2012) Connecting mTORC1 signaling to SREBP-1 activation. Curr Opin Lipidol 23: 226–234. 22449814 10.1097/MOL.0b013e328352dd03

[18] Chen WQ, Siegel N, Li L, Pollak A, Hengstschlager M, et al (2009) Variations of protein levels in human amniotic fluid stem cells CD117/2 over passages 5–25. J Proteome Res 8: 5285–5295. 19791749 10.1021/pr900630s

[19] Swoboda A, Schanab O, Tauber S, Bilban M, Berger W, et al (2012) MET expression in melanoma correlates with a lymphangiogenic phenotype. Hum Mol Genet 21: 3387–3396. 22570180 10.1093/hmg/dds171

[20] Schneider CA, Rasband WS, Eliceiri KW (2012) NIH Image to ImageJ: 25 years of image analysis. Nat Methods 9: 671–675. 22930834 10.1038/nmeth.2089 PMC5554542

[21] Fuchs C, Rosner M, Dolznig H, Mikula M, Kramer N, et al (2012) Tuberin and PRAS40 are anti-apoptotic gatekeepers during early human amniotic fluid stem-cell differentiation. Hum Mol Genet 21: 1049–1061. 22090422 10.1093/hmg/ddr535

[22] Siegel N, Rosner M, Unbekandt M, Fuchs C, Slabina N, et al (2010) Contribution of human amniotic fluid stem cells to renal tissue formation depends on mTOR. Hum Mol Genet 19: 3320–3331. 20542987 10.1093/hmg/ddq236

[23] Calandra S, Tarugi P, Speedy HE, Dean AF, Bertolini S, et al (2011) Mechanisms and genetic determinants regulating sterol absorption, circulating LDL levels, and sterol elimination: implications for classification and disease risk. J Lipid Res 52: 1885–1926. 21862702 10.1194/jlr.R017855 PMC3284125

[24] King RH, Chandler D, Lopaticki S, Huang D, Blake J, et al (2011) Ndrg1 in development and maintenance of the myelin sheath. Neurobiol Dis 42: 368–380. 21303696 10.1016/j.nbd.2011.01.030

[25] Bilheimer DW, Grundy SM, Brown MS, Goldstein JL (1983) Mevinolin and colestipol stimulate receptor-mediated clearance of low density lipoprotein from plasma in familial hypercholesterolemia heterozygotes. Proc Natl Acad Sci U S A 80: 4124–4128. 6575399 10.1073/pnas.80.13.4124 PMC394213

[26] Schalm SS, Tee AR, Blenis J (2005) Characterization of a conserved C-terminal motif (RSPRR) in ribosomal protein S6 kinase 1 required for its mammalian target of rapamycin-dependent regulation. J Biol Chem 280: 11101–11106. 15659381 10.1074/jbc.M413995200

[27] Schalm SS, Blenis J (2002) Identification of a conserved motif required for mTOR signaling. Curr Biol 12: 632–639. 11967149 10.1016/s0960-9822(02)00762-5

[28] Caddick J, Kingham PJ, Gardiner NJ, Wiberg M, Terenghi G (2006) Phenotypic and functional characteristics of mesenchymal stem cells differentiated along a Schwann cell lineage. Glia 54: 840–849. 16977603 10.1002/glia.20421

[29] Fu Q, Goodrum JF, Hayes C, Hostettler JD, Toews AD, et al (1998) Control of cholesterol biosynthesis in Schwann cells. J Neurochem 71: 549–555. 9681444 10.1046/j.1471-4159.1998.71020549.x

[30] Wegner M (2000) Transcriptional control in myelinating glia: flavors and spices. Glia 31: 1–14. 10816602 10.1002/(sici)1098-1136(200007)31:1<1::aid-glia10>3.0.co;2-v

[31] Heller BA, Ghidinelli M, Voelkl J, Einheber S, Smith R, et al (2014) Functionally distinct PI 3-kinase pathways regulate myelination in the peripheral nervous system. J Cell Biol 204: 1219–1236. 24687281 10.1083/jcb.201307057 PMC3971744

[32] Sheean ME, McShane E, Cheret C, Walcher J, Muller T, et al (2014) Activation of MAPK overrides the termination of myelin growth and replaces Nrg1/ErbB3 signals during Schwann cell development and myelination. Genes Dev 28: 290–303. 24493648 10.1101/gad.230045.113 PMC3923970

[33] Chrast R, Saher G, Nave KA, Verheijen MH (2011) Lipid metabolism in myelinating glial cells: lessons from human inherited disorders and mouse models. J Lipid Res 52: 419–434. 21062955 10.1194/jlr.R009761 PMC3035679

[34] Boyles JK, Zoellner CD, Anderson LJ, Kosik LM, Pitas RE, et al (1989) A role for apolipoprotein E, apolipoprotein A-I, and low density lipoprotein receptors in cholesterol transport during regeneration and remyelination of the rat sciatic nerve. J Clin Invest 83: 1015–1031. 2493483 10.1172/JCI113943 PMC303779

[35] Zhao S, Hu X, Park J, Zhu Y, Zhu Q, et al (2007) Selective expression of LDLR and VLDLR in myelinating oligodendrocytes. Dev Dyn 236: 2708–2712. 17685481 10.1002/dvdy.21283

[36] Maier O, De Jonge J, Nomden A, Hoekstra D, Baron W (2009) Lovastatin induces the formation of abnormal myelin-like membrane sheets in primary oligodendrocytes. Glia 57: 402–413. 18814266 10.1002/glia.20769

[37] Murinson BB, Haughey NJ, Maragakis NJ (2012) Selected statins produce rapid spinal motor neuron loss in vitro. BMC Musculoskelet Disord 13: 100. 22703530 10.1186/1471-2474-13-100 PMC3487793

[38] Paintlia AS, Paintlia MK, Khan M, Vollmer T, Singh AK, et al (2005) HMG-CoA reductase inhibitor augments survival and differentiation of oligodendrocyte progenitors in animal model of multiple sclerosis. FASEB J 19: 1407–1421. 16126908 10.1096/fj.05-3861com

[39] Peterson TR, Sengupta SS, Harris TE, Carmack AE, Kang SA, et al (2011) mTOR complex 1 regulates lipin 1 localization to control the SREBP pathway. Cell 146: 408–420. 21816276 10.1016/j.cell.2011.06.034 PMC3336367

[40] Myckatyn TM, Ellis RA, Grand AG, Sen SK, Lowe JB 3rd, et al (2002) The effects of rapamycin in murine peripheral nerve isografts and allografts. Plast Reconstr Surg 109: 2405–2417. 12045568 10.1097/00006534-200206000-00035

