# Supplementary figures and images for: mTORC1 Is Essential for Early Steps during Schwann Cell Differentiation of Amniotic Fluid Stem Cells and Regulates Lipogenic Gene Expression
Source: PLoS One. 2014 Sep 15;9(9):e107004. doi: 10.1371/journal.pone.0107004 (PMC4164523; doi:10.1371/journal.pone.0107004)

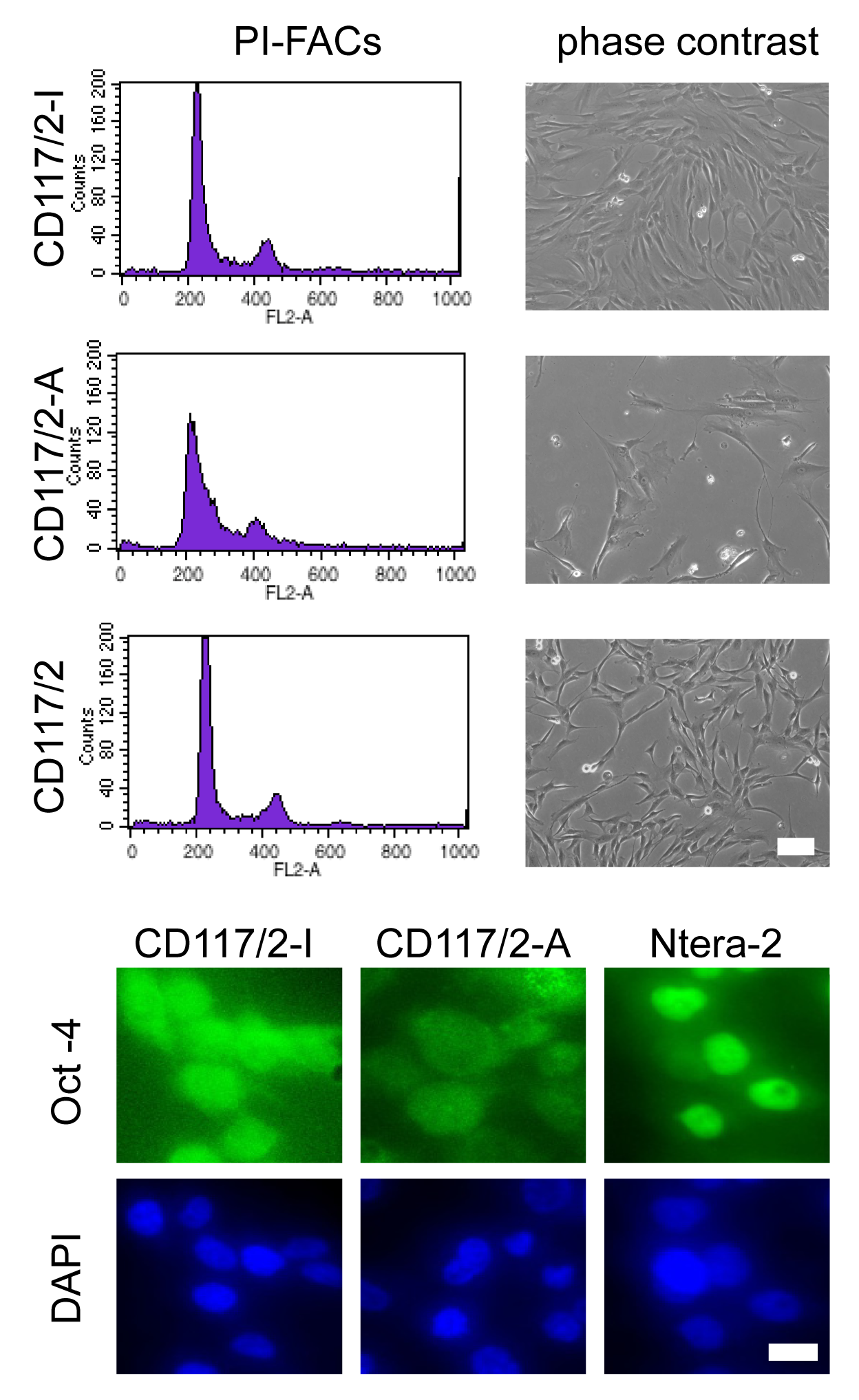

Supplement: Figure S1 — CD117/2 amniotic fluid stem cells were single cell cloned by limiting dilution. Single cell clone CD117/2-I displayed a normal propidium iodide stain, as observed in the starting population CD117/2. CD117/2-I exhibited a small and uniform cell morphology characteristic for bona fide amniotic fluid stem cells. Immunofluorescence staining with the santa cruz antibody sc-5789 revealed a strong nuclear Oct-4 stain in CD117/2-I cells similar to Ntera-2 carcinoembryonal cells used as control cells. Single cell clone CD117/2-A is shown as an unsuitable cell line, which displayed abnormal propidium iodide stain, heterogeneous and large cells in culture and no Oct-4 stain. Scale bar represents 5 µm. PI-FACs = propidium iodide fluorescence activated cell scanning. (TIF) [file pone.0107004.s001.tif]

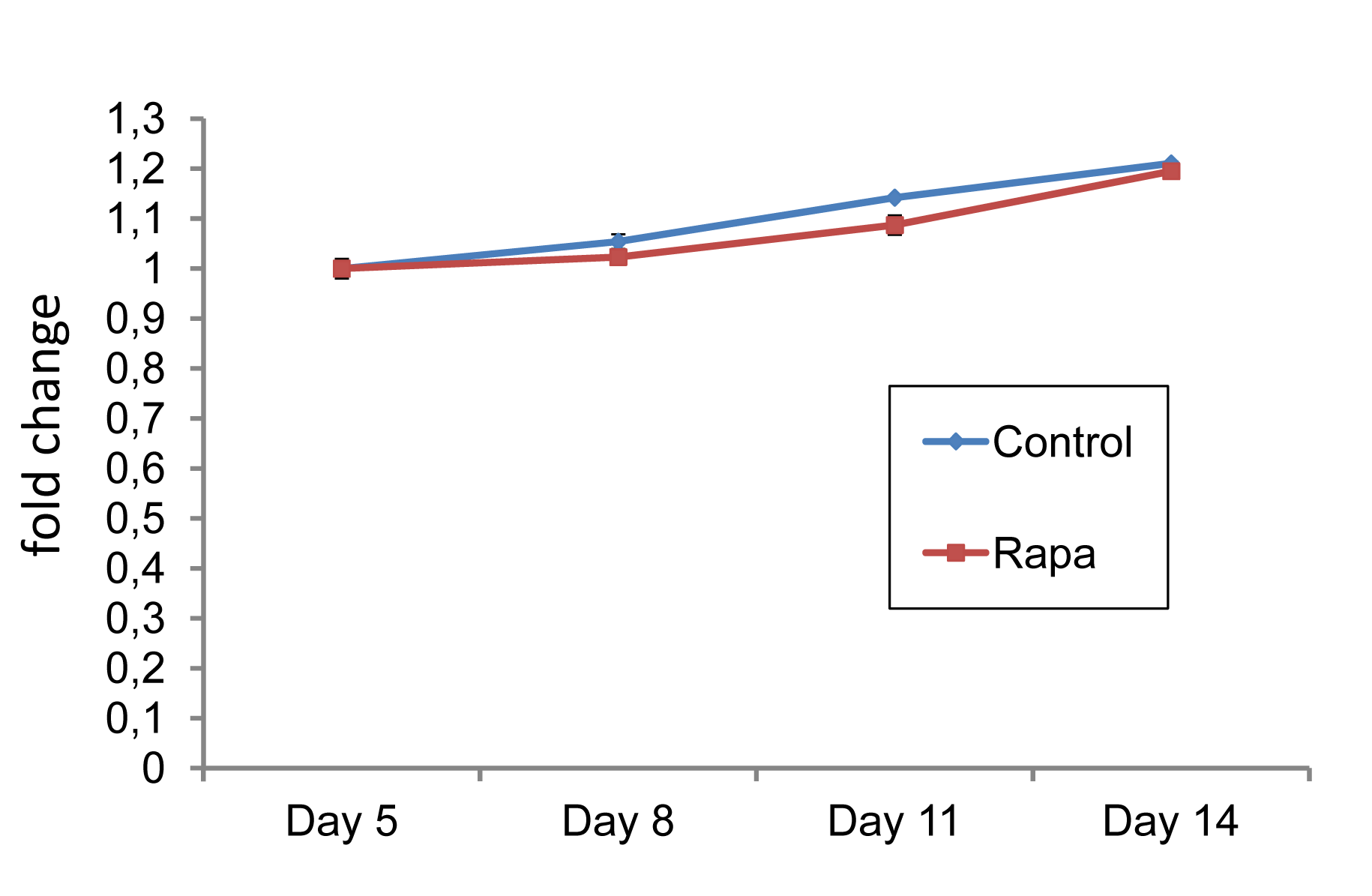

Supplement: Figure S2 — Starting from day 5 of differentiation the effect of 25 nM rapamycin on cell viability was measured by using Alamar blue. Cells were seeded equally on day 5 of differentiation and continued to be treated with differentiation media III (see Fig. 1) either with or without the addition of rapamycin. Alamar blue was added and cells were incubated for an additional 4 hours. The fluorescence was measured at wavelengths excitation 540 nm and emission 590 nm. The average out of 4 measurments is shown +/− S.D. (TIF) [file pone.0107004.s002.tif]

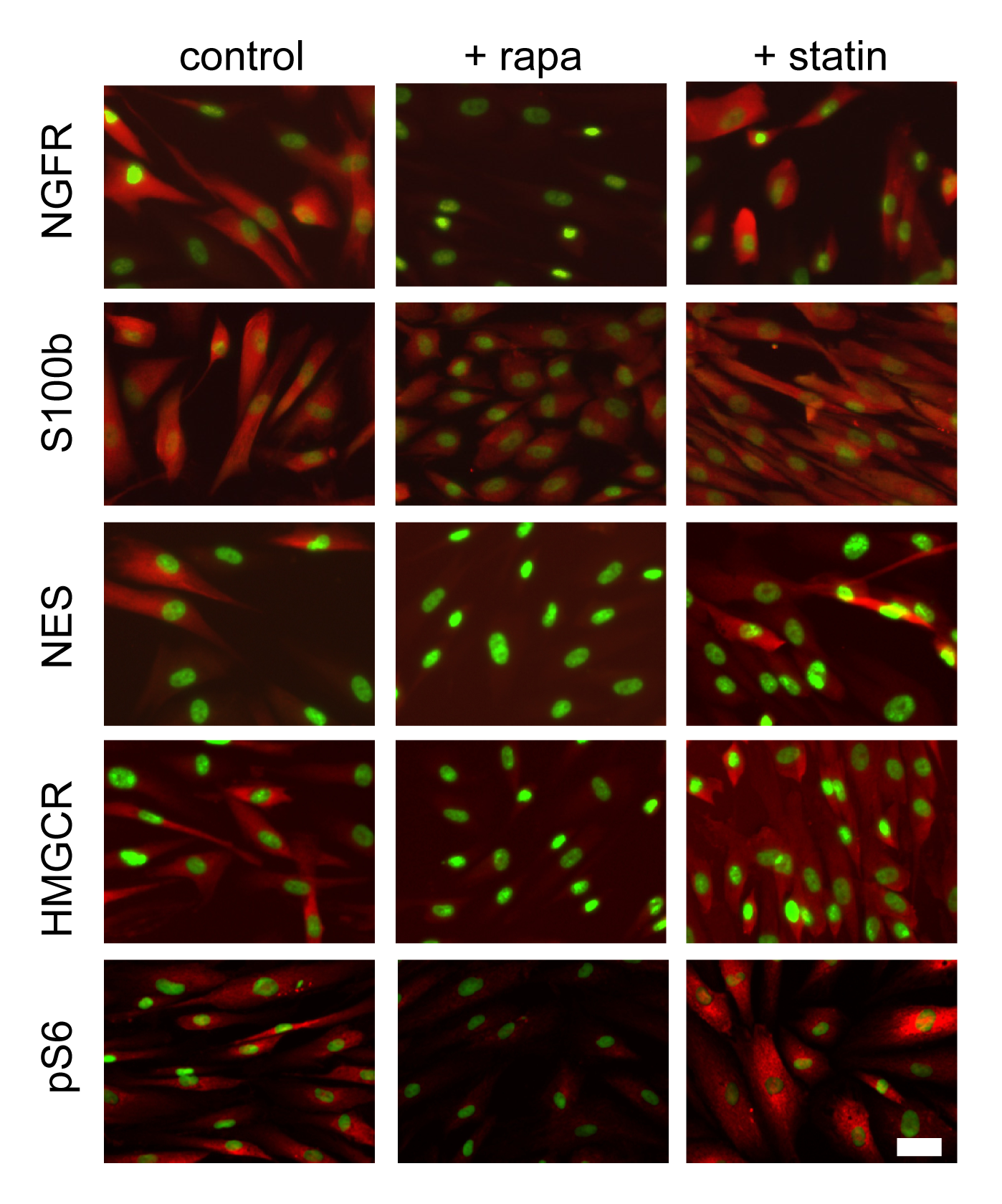

Supplement: Figure S3 — AFS cells were differentiated for 15 days and continuously treated either with 25 nM rapamycin or 1 µM of statin. Fixed cells were stained with indicated antibodies (labeled in red, nuclei in green). Scale bar represents 10 µm. (TIF) [file pone.0107004.s003.tif]

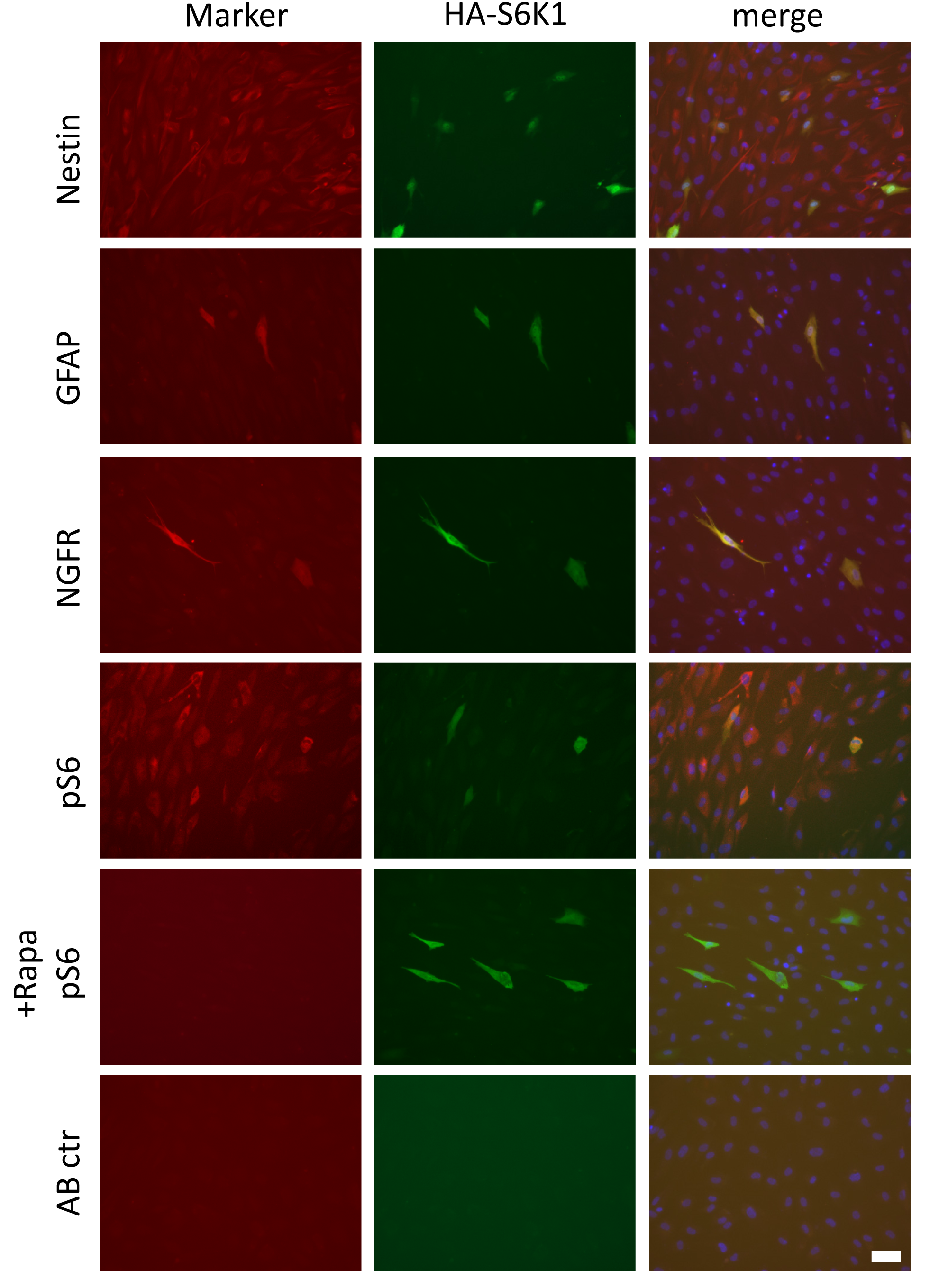

Supplement: Figure S4 — AFS cells were differentiated as described in material and methods and at day 15 cells were transfected with an HA-fused wild type S6K1 (HA-S6K1) purchased from Addgene. After 72 hours in differentiation media cells were fixed and stained with anti-HA antibody (shown in green) combined with antibodies detecting Nestin, GFAP, NGFR and phosphorylated S6 (shown in red). Rapa = Rapamycin treatment for 72 hours. AB ctr = antibody control stain. Scale bar represents 25 µm. (TIF) [file pone.0107004.s004.tif]

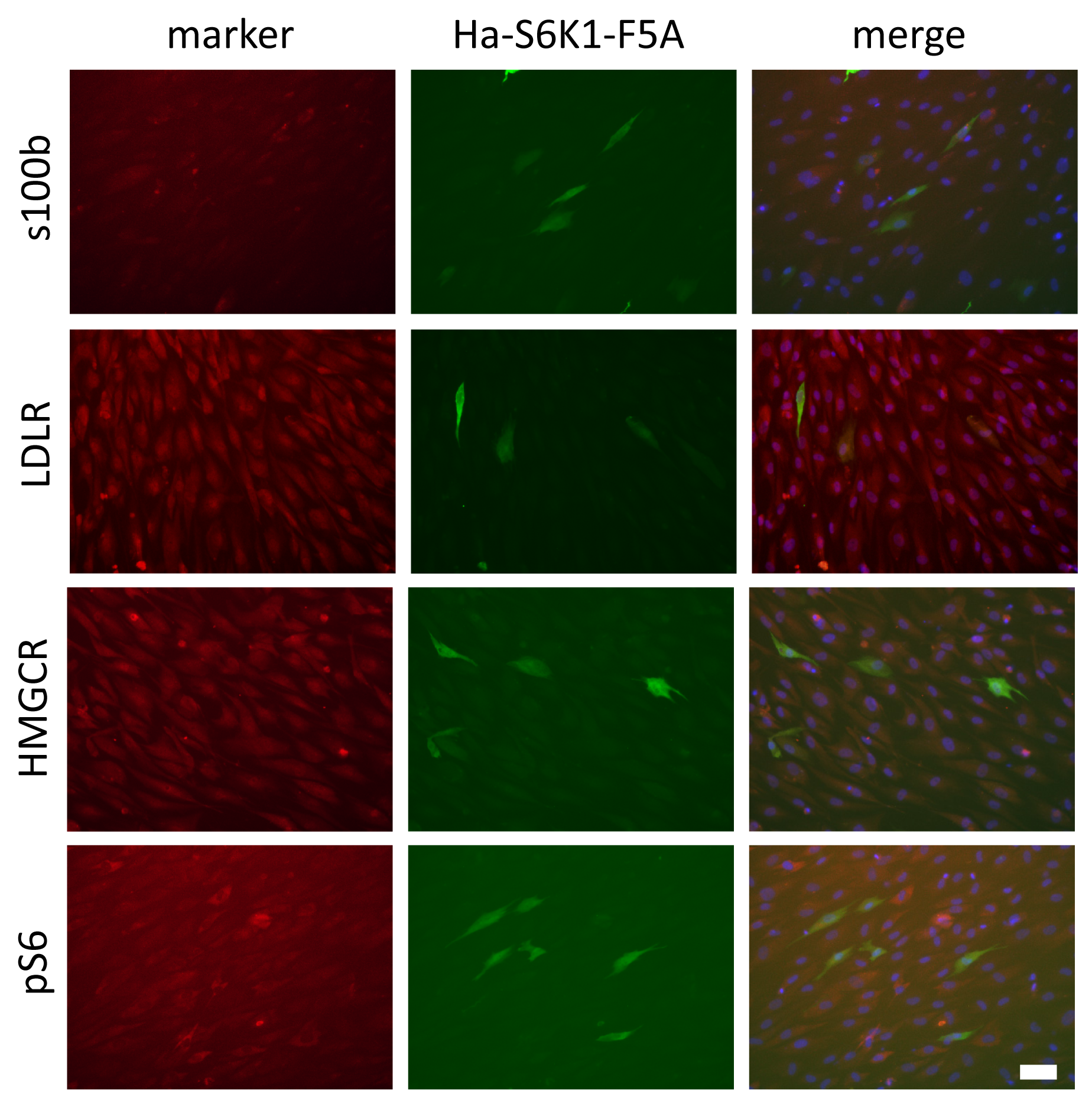

Supplement: Figure S5 — AFS cells were differentiated as described in material and methods and at day 15 cells were transfected with an HA-fused TOS motive mutated S6K1 (HA-S6K1-F5A), purchased from Addgene. After 72 hours in differentiation media cells were fixed and stained with anti-HA antibody (shown in green) combined with antibodies detecting S100b, LDLR, HMGCR and phosphorylated S6 (shown in red). Rapa = Rapamycin treatment for 72 hours. AB ctr = antibody control stain. Scale bar represents 25 µm. (TIF) [file pone.0107004.s005.tif]
